# Block Copolymer-Templated, Single-Step Synthesis of Transition Metal Oxide Nanostructures for Sensing Applications

**DOI:** 10.1021/acsami.3c10439

**Published:** 2023-08-29

**Authors:** Przemyslaw Pula, Arkadiusz A. Leniart, Julia Krol, Maciej T. Gorzkowski, Mihai C. Suster, Piotr Wrobel, Adam Lewera, Pawel W. Majewski

**Affiliations:** † Department of Chemistry, University of Warsaw, Warsaw 02093, Poland; ‡ Biological and Chemical Research Centre, University of Warsaw, Warsaw 02089, Poland; § Department of Physics, University of Warsaw, Warsaw 02093, Poland

**Keywords:** block copolymers, directed self-assembly, solvent evaporation annealing, transition metal oxide nanowires, gas sensors

## Abstract

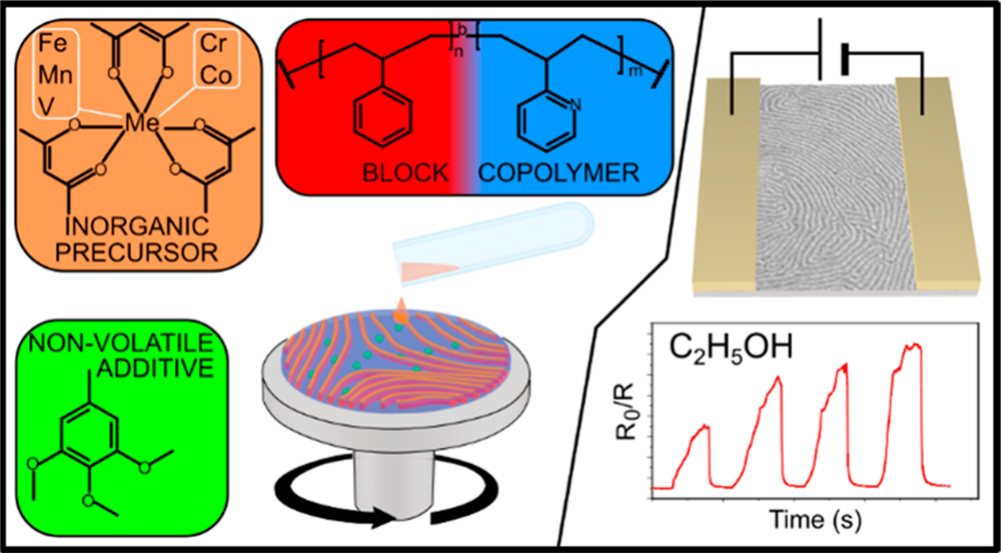

The synthesis of transition metal oxide nanostructures, thanks to their high surface-to-volume ratio and the resulting large fraction of surface atoms with high catalytic activity, is of prime importance for the development of new sensors and catalytic materials. Here, we report an economical, time-efficient, and easily scalable method of fabricating nanowires composed of vanadium, chromium, manganese, iron, and cobalt oxides by employing simultaneous block copolymer (BCP) self-assembly and selective sequestration of metal–organic acetylacetonate complexes within one of the BCP blocks. We discuss the mechanism and the primary factors that are responsible for the sequestration and conformal replication of the BCP template by the inorganic material that is obtained after the polymer template is removed. X-ray photoelectron spectroscopy (XPS) and powder X-ray diffraction (PXRD) studies indicate that the metal oxidation state in the nanowires produced by plasma ashing the BCP template closely matches that of the precursor complex and that their structure is amorphous, thus requiring high-temperature annealing in order to sinter them into a crystalline form. Finally, we demonstrate how the developed nanowire array fabrication scheme can be used to rapidly pattern a multilayered iron oxide nanomesh, which we then used to construct a prototype volatile organic compound sensor.

## Introduction

Sustained progress in the development of modern electronics relies on a constant pursuit of their miniaturization, reducing their energy usage, and simplifying their fabrication. The wide selection of economically viable alternatives to conventional top-down-produced counterparts is critical for new-generation devices that are applicable in the dynamically developing fields of smart household appliances, autonomous vehicles, and wearable electronics. Chemical sensors will likely be an integral component of such devices, as they are critical for the detection and assessment of potential environmental hazards. While designing an efficient gas sensing material, one should consider its structural parameters, such as a high surface area-to-volume ratio of the active layer, and its chemical composition to ensure the optimal sensing output.^1,2^ Nanostructured porous materials instantly fulfill the first criterion, but their widespread implementation relies on the development of scalable fabrication methods, such as those offered by the self-assembly of block copolymers (BCPs). This prominent class of organic macromolecules consisting of distinct polymer chains possesses a characteristic ability to microphase-separate and self-assemble into different nanoscale morphologies such as cylinders, lamellae, or gyroids.^3^ Conveniently, nanoscale BCP morphologies can be further used as synthetic templates to pattern a variety of functional inorganic nanostructures^4^ for their further use in micro-electronic elements,^5^ photonics,^6^ molecular separations,^7^ and energy harvesting^8^ applications.

BCP self-assembly can be induced and controlled in several ways. Thermal annealing of a polymer above its glass transition temperature is perhaps the simplest BCP ordering method, yet it frequently takes a prohibitively long time due to the risk of thermal degradation when using high ordering temperatures.^9,10^ However, this problem can be significantly mitigated by applying rapid transient heating ramps, which are enabled by irradiation with high-power laser beams^11^ or infrared (IR) lamps.^12−14^ BCP self-assembly can be greatly accelerated by utilizing one of the more sophisticated techniques from the arsenal of directed self-assembly (DSA) methods. These methods utilize electric,^15^ magnetic,^16^ or shearing^17^ fields, as well as graphoepitaxial^18,19^ or chemoepitaxial^20^ interactions, to overcome the kinetic barriers of spontaneous self-assembly and shorten the ordering time. A popular technique of solvent-assisted BCP ordering called solvent vapor annealing (SVA, i.e., BCP ordering in the presence of a plastifying solvent vapor) can also significantly improve and accelerate the ordering.^21^ An even simpler preparation of the BCP matrix is offered by solvent evaporation annealing (SEA)^22,23^ and other simultaneous casting-and-ordering methods.^24,25^ In this kind of method, the microphase separation of the BCP domains occurs during the prolonged evaporation of a nonvolatile solvent or a mixture of two nonvolatile and volatile co-solvents, which facilitates BCP ordering during the casting step. As a result, large-grained morphologies (i.e., ordered arrays of characteristic BCP motifs with long-range spatial coherence) form during the effortless single-step, casting-and-ordering process. Therefore, such methods have great prospects in terms of their use for large-scale production of ordered BCP morphologies for patterning applications, provided that the safety issues connected with the use of organic solvents are resolved.

The templated synthesis of inorganic nanostructures with BCP matrices is usually a multistep process. It starts with preparing an ordered BCP thin film (i.e., the template) using one of the DSA methods. This step is followed by the selective infusion of an inorganic precursor into one of the BCP blocks or its selective removal and subsequent deposition of the inorganic material in the created voids. Recent reviews of the most popular BCP-templated synthesis techniques have been provided by Cummins et al.^26^ and Subramanian et al.^27^ BCP infusion techniques include vapor phase sequential infiltration synthesis (SIS), which utilizes volatile organometallic precursors that display preferential absorption to one of the blocks that, after conversion, results in metal oxide nanostructures (such as Al_2_O_3_, ZnO, or TiO_2_).^28−30^ A complementary approach, wet chemical conversion, employs the absorption of aqueous metal complexes and their coordination with poly(vinylpyridine) blocks, yielding metallic Pt, Au, Cu, Co, and Ni nanopatterns.^31−33^ Unlike these additive infusion methods, one of the blocks of a BCP template can be selectively removed by chemical etching and backfilled (e.g., by electrodeposition) with an inorganic material. This approach, which requires conductive support of the BCP films, has been demonstrated for a range of metals and BCP morphologies.^34−36^ An even simpler method for generating metallic or oxide nanostructure templates has been proposed by Ghoshal et al., who spin-coated metal salt solutions over pre-ordered BCP films that contained a poly(ethylene oxide) (PEO) block as a host for inorganic salts and demonstrated the synthesis of various nanopatterns containing iron, copper, titanium, and cobalt oxides.^37,38^ A similar method that also used PEO as a hosting block was explored by Xu et al. to fabricate arrays of cobalt ferrite nanodots.^39^

The idea of including inorganic precursor species in the block copolymer casting solution has been investigated for various amphiphilic BCP systems (i.e., those that contain at least one block that is capable of hosting polar precursor species). Russell et al. investigated the formation of nanostructured titania by concurrent BCP ordering and the selective decoration of PEO domains with TiO_2_ while casting a BCP solution containing titanium isopropoxide.^40^ Zhang et al. blended triblock terpolymer templates with resorcinol-phenol resin to yield mesoporous carbon replicas after subsequent SVA and pyrolysis.^41^ Analogously, He et al. used the calcination of tetraethyl orthosilicate-infused BCP to produce mesostructured silicas.^42^ An alternative approach to ordered arrays of inorganic nanomaterials consists of blending presynthesized nanoparticles (NPs) with the BCP casting solution; such an approach has been demonstrated for iron oxide,^43,44^ Au,^45^ PbS,^46^ and CdSe^46,47^ NPs. Müller-Buschbaum et al. explored the synthesis of transition metal oxide nanostructures (TiO_2_, ZnO, SnO_2_, and Fe_2_O_3_) using the polystyrene-*block*-poly(ethylene oxide) (PS-*b*-PEO)^48,49^ and polystyrene-*block*-poly(4-vinylpyridine) (PS-*b*-P4VP)^50,51^ BCPs, monitoring the hybrid-film formation with *in situ* grazing-incidence small-angle X-ray scattering (GISAXS) measurements.^51^

Potential drawbacks of directly blending the inorganic precursor with the BCP are limited metal loading and the risk of disturbing the targeted BCP morphology at a too-high precursor content.^52^ Additionally, in the case of reactive precursors, metallic species can adversely impact the chain mobility of the BCP by their complexation and chemical cross-linking, which results in defective nanostructures that have a low degree of lateral order (i.e., small-grained morphologies).^51^

In this work, we describe a universal synthesis pathway for fabricating inorganic thin-film nanostructures composed of first-row transition metal oxides (V, Cr, Mn, Fe, and Co), replicating the morphology of the PS-*b*-P2VP templates. The ordering of the BCP templates and the selective deposition of the nonpolar transition metal precursors (Me(III) acetylacetonates) occurs during a single spin-casting step. This is realized by a methodical adjustment of the composition of the casting solution containing the block copolymer, the mixture of volatile and nonvolatile solvents, and the highly soluble acetylacetonate metal precursors. The optimal composition of the casting solution, as well as the proper adjustment of the casting parameters, leads to long-range ordered BCP morphologies with a selectively infused P2VP block and, after the subsequent plasma ashing step, to conformal metal oxide nanopatterns. As a proof-of-concept and a demonstration of the utility of our method, we manufactured a multilayered porous metal oxide nanomesh and used it as an active layer in an ethanol vapor sensor.

## Experimental Section

### Materials

Cylinder-forming polystyrene-*block*-poly(2-vinylpyridine) (PS-*b*-P2VP) block copolymers with the following compositions of 79.0 kg mol^–1^-*b*-36.5 kg mol^–1^ (PDI = 1.05), abbreviated as C116, and 185 kg mol^–1^-*b*-90 kg mol^–1^ (PDI = 1.10), abbreviated as C275, were purchased from Polymer Source. Anhydrous acetylacetonate salts of iron(III), cobalt(III) chromium(III), manganese(III), and vanadium(III) were purchased from Sigma-Aldrich and dissolved in GPC-grade toluene (Carl Roth) to yield 5% w/w stock solutions.

### Thin-Film Casting

Standard, single-sided polished, electronic-grade Si wafers with thicknesses of ∼500 μm that were purchased from ITME, Poland, were used as polymer substrates. The C116 and C275 BCPs were dissolved in dry toluene with molecular sieve-dried co-solvents: 3,4-dimethoxytoluene (DMOT; 97%, TCI Chemicals) or 3,4,5-trimethoxytoluene (TMOT; 97%, Sigma-Aldrich). They were then mixed with an acetylacetonate trivalent metal stock solution at the appropriate molar ratio of metal to vinylpyridine (e.g., 1:4, 1:2, and 1:1) to yield the final polymer concentration of 1% and 1.5% for C116 and C275, respectively. The solutions were filtered with a 0.2 μm PTFE syringe filter before use. The silicon substrates (15 mm × 15 mm) were briefly cleaned with oxygen plasma (PE-25, Plasma Etch; 150 mTorr O_2_, 100 W of radio frequency (RF) power, 180 s) immediately before spin-coating was performed at room temperature for 300 s (SPIN150i, SPS-Europe), where the spin-coater chamber was under a constant supply of dry air (∼10 L/min). A spin-coating rate adjustment (2000–6000 rpm) was used to control the final thickness of the dried films, as verified by white light spectral reflectometry (Filmetrics F20 UV, KLA Instruments) and ellipsometry (Nanofilm EP3, Accurion GmbH).

### Evaporation Rate Control

We followed a protocol that has been established previously.^22^ The samples, after being spin-coated with TMOT-containing solutions, were transferred onto a preheated metal plate to finish solvent evaporation at an elevated temperature (40–80 °C). For controlling the evaporation rate, we introduced aluminum caps with perforations that functioned as convective evaporation limiters. The caps were positioned over the drying samples, following the approach employed in our previous study.^22^

### Sensor Fabrication

A ∼1 μm thick silicon dioxide insulating layer was thermally grown on a silicon substrate (1200 °C, chamber furnace, Carbolite Gero GmbH). Next, the Fe(acac)_3_-infused C116 solution (2% C116 in 20% DMOT–toluene with a Fe:VP ratio of 1:2) was spin-coated at 2000 rpm at room temperature to form a film with a thickness of ∼100 nm. After this step, the sample was briefly dried at 120 °C to remove any excess solvent before the oxygen plasma BCP ashing step (150 mTorr of O_2_, 100 W of RF power, 600 s). The same procedure was repeated four times to obtain an iron oxide nanostructured mesh. Then, thermal annealing in dry air (550 °C, 2 h) was performed to improve the degree of crystallinity of the material, reducing the final thickness of the mesh to ∼120 nm (measured by cross-sectional scanning electron microscopy). Six interdigitated electrodes (400 μm long by 100 μm wide with a 50 μm gap) were patterned in a positive photoresist (AZ 1514H, Microchemicals GmbH) using a maskless laser writing method and washed with a developer (AZ 726 MIF, Microchemicals GmbH). After evaporation of the 70 nm thick Au electrodes (nanoPVD-T15A, Moorfield Nanotechnology), the undeveloped resist was removed by liftoff using 1-methyl-2-pyrrolidone (99.5%, anhydrous; Sigma-Aldrich) at 40 °C. Finally, the organic residues were removed by being immersed in acetone with mild sonic agitation and then rinsed with ultrapure water.

### Sensor Testing

The sensor’s response to ethanol vapor was examined with an electrical probe station with a hermetic chamber (HCP622G-PS, Instec Inc.), equipped with a heating station and a source measure unit (2450, Keithley Instruments) that operated in a two-wire configuration. Ethanol vapor was produced by passing a stream of dry nitrogen through a bubbler filled with anhydrous alcohol, which was further diluted with a stream of pure N_2_ in a custom-made gas mixer before being delivered to the chamber. The reference ethanol concentration was measured with a commercial gas sensor (SGP30, Sensirion AG; declared accuracy 15%) and adjusted by regulating the flow of the EtOH-rich N_2_ stream with a needle valve.

### Plasma Ashing

Oxygen plasma etching was used to remove the polymer matrix and decompose the acetylacetonate metal precursors (PE-25, Plasma Etch; 150 mTorr of O_2_, 100 W of RF power, 1200 s).

### Scanning Electron Microscopy

The plasma-etched samples were examined under a field emission scanning electron microscope (Zeiss Merlin), operating at 3 keV and equipped with an in-lens detector for secondary electrons.

### Powder XRD

Acetylacetonates of the respective metals that were dissolved in ethanol or a BCP–toluene solution were cast on silicon substrates and heated to the specified annealing temperature. Diffractograms were collected *in situ* during thermal annealing using a powder X-ray diffractometer (D8 DISCOVER, Bruker) with collimated Cu Kα radiation (0.154 nm) in the 20–70° 2θ range using the locked-coupled mode. Data fitting and analysis were performed using the TOPAS software (Bruker).

### XPS Analysis

For X-ray photoelectron spectroscopy (XPS) measurements, we prepared thin films of PS-*b*-P2VP C275 with 1:2 Me:VP loading. For each metal (Fe, Co, Mn, Cr, V), 4 samples were prepared: (1) an Me(acac)_3_-infused BCP film, (2) a sample ashed in O_2_ plasma for 30 min, (3) a sample ashed in air at 600 °C in a muffle furnace for 1 h (Czylok FCF5), and (4) a sample annealed for 5 min at 600 °C in an Ar/H_2_ atmosphere in a rapid thermal processing setup with a 70 W IR heat source. The presence of adstructures that formed on the surface of the samples, which is responsible for undesirable charging, was corrected by careful energy scale calibration (further details can be found in the Supporting Information).

XPS spectra were measured using a SPECS Surface Nano Analysis GmbH (Berlin, Germany) instrument with an XR 50 X-ray source that uses a non-monochromatic X-ray Al Kα emission line (with a photon energy of 1486.6 eV and operating at 270 W) and a non-monochromatic Mg Kα emission line (with a photon energy of 1253.64 eV and operating at 180 W). The XP spectrometer was equipped with a PHOIBOS 150 NAP hemispherical analyzer (150 mm radius) with a 2D-DLD detector. To clean the metal samples, Ar ion etching was performed, for which an IQE 12/38 sputter gun (SPECS Surface Nano Analysis GmbH, Berlin, Germany) and gaseous argon (99.9999%, O_2_ < 0.01 ppm) were utilized. The ion etching procedures were performed in the analysis chamber of the XP spectrometer. The system base pressure was in the 1 × 10^–10^ mbar range, and no charge compensation was used. The XPS spectra were fitted using the CasaXPS software, version 2.3.24PR1.0.^53^ Iron, cobalt, chromium, and manganese sputtering targets (99.95% purity; Kurt J. Lesker Co.) as well as vanadium (99.8%; Goodfellow) were used. Oxidized metal references were obtained by exposing the metallic substrates to air; the XPS spectra of these were collected without prior cleaning with Ar ion bombardment.

## Results and Discussion

### Single-Step Casting Parameters and Morphology Characterization

**Figure 1:**
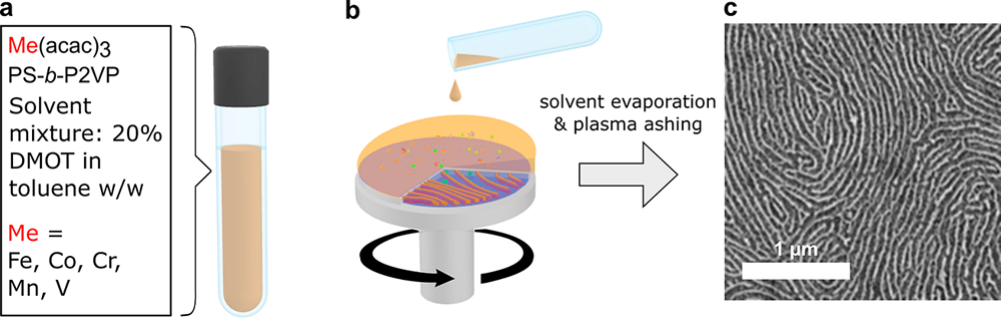
Schematic of the single-step rapid fabrication of metal oxide nanostructures using BCP templating. (a) The composition of the casting solution, (b) simultaneous spin-casting and ordering, and (c) an SEM image of the iron oxide nanowires that were obtained by casting Fe(acac)_3_/C116 with a 1:2 Fe:VP ratio from the 20% DMOT–toluene mixture followed by O_2_ plasma ashing.

We initially examined the solubility of several bi- and trivalent transition metal compounds in the previously studied BCP casting mixture, which facilitates the rapid ordering of cylindrical PS-*b*-P2VP during casting.^22^ The mixture contains a volatile base solvent (toluene) and one of the nonvolatile co-solvents DMOT or TMOT, which prolong the evaporation process and enable BCP ordering.^22^ Among the tested salts of the first-row transition metals, including acetates, phthalates, and acetylacetonates, only the anhydrous trivalent metal acetylacetonates of V, Cr, Mn, Fe, and Co displayed sufficient solubility in the casting mixture to be used for further patterning, thanks to the nonpolar nature. We prepared stock solutions of BCPs and Me(acac)_3_ in the 20% DMOT or 10% TMOT mixture at 2% and 5% (w/w), respectively. These solutions were mixed at the appropriate molar ratio of the Me(III) precursor to the 2-vinylpyridine monomeric units in the P2VP block of the BCP chain (termed Me:VP) and diluted to the final BCP concentration of 1 wt %. In this study, the mixing was performed immediately before spin-casting the thin films onto the Si substrates. We note, however, that the Me(III) acetylacetonate complexes are remarkably stable against pyridine ligand exchange in the solution, except for vanadium and (to a lesser extent) manganese, whose UV–vis spectra are altered after 24 h of exposure to pyridine in 10:1 molar excess (Figure S1). Therefore, the casting solutions can be used for an extended period. Conversely, it is critical to use anhydrous materials; the prolonged storage of solutions in open containers in a moist environment results in irreproducible patterning. The schematic concept of the single-step metal oxide nanowire (NW) patterning is shown in Figure 1. The typical multistep process of BCP template preparation and annealing, followed by the infusion of the metal precursor,^32,54^ is reduced to a single casting step by using a solution containing BCP and the metal precursor salt (Figure 1a), which only requires basic lab equipment and mere minutes to cast (Figure 1b). The morphology of a thin-film sample that was derived from a blend of Fe(acac)_3_ and cylindrical PS-*b*-P2VP of 116 kg mol^–1^ (C116) using a 1:2 Fe:VP molar ratio, which was cast on a Si substrate after oxygen plasma removal of the BCP matrix, is shown in Figure 1c. The sample presents a well-ordered horizontal cylinder morphology that largely resembles the neat diblock that was processed under similar conditions (Figure S2). The complete evaporation of the solvent mixture (i.e, spin-to-dry) takes approximately 3 min.

### Stoichiometry

**Figure 2:**
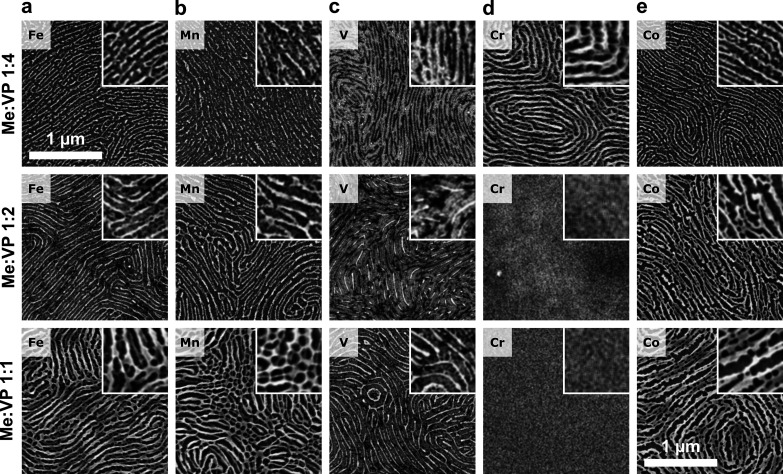
SEM morphologies of transition metal oxide nanostructures derived from cylindrical PS-*b*-P2VP of 275 kg mol^–1^ (C275) blended with (a) iron(III), (b) manganese(III), (c) vanadium(III), (d) chromium(III), and (e) cobalt(III) acetylacetonates at various Me:VP stoichiometric ratios. All of the samples were prepared by spin-casting from a 1.5% BCP/10% TMOT–toluene mixture at room temperature. The insets span 400 × 400 nm.

We prepared a series of casting solutions containing a higher molecular weight (275 kg mol^–1^, termed C275) homologue of the C116 and transition metal complexes at various molar ratios ranging from 1:4 to 1:1 Me:VP metal loading. The solutions containing 1.5% BCP (w/w) were all cast at 2000 rpm onto freshly cleaned Si substrates until dry films were obtained. The films were subsequently oxygen plasma ashed and examined under SEM, revealing inorganic nanostructured patterns. At the lowest metal loading (1:4), the patterns closely resemble the nanowires that are characteristic of cylinder-forming BCP templates that have the inorganic material sequestered into the minority P2VP block. This metal loading level is, however, insufficient for yielding continuous nanowires in the case of the Fe-, Mn-, and Co-bearing nanostructures (Figure 2a,b,e; top row). For these metals, the optimal precursor loading for yielding single-layer arrays of ordered nanowires is close to 1:2 (Figure 2a,b,e; middle row), though some “branching” is visible in the case of Co. At a 1:1 Me:VP ratio, multilayered (Fe, Mn, V), highly branched (Co), and mesh (Mn) nanostructures form. The first type of nanostructure, which was also observed in the C116 homologue-patterned NWs of Cr (with a 1:2 Me:VP ratio) and Mn and V (with a 1:1 Me:VP ratio) (Figure S3), bears a resemblance to a perforated lamellar morphology^55^ and is likely a result of a high degree of swelling of the minority block with the acetylacetonate precursor. The morphology–metal loading trend that is observed for the nanostructures derived from chromium(III) acetylacetonate differs from that of the other metals (Figure 2d). While continuous horizontal nanowires are observed at the 1:4 metal loading, there is an apparent disappearance of the nanostructure features at the 1:1 Cr(acac)_3_ loading, both in C275 and in C116. We ascribe this to the formation of a continuous inorganic surface during plasma ashing that is likely caused by the nonselective sequestration of the precursor near the surface of the BCP film. On the level of molecular interactions, this trend can be explained by the stronger binding energy of the acac ligand and chromium(III) compared to that of the iron complex^56^ and the higher thermal stability of chromium acetylacetonate compared to that of the corresponding Mn, Fe, or Co salts,^57^ which lowers the propensity for interactions of Cr with the pyridine units that are present in the P2VP block.

### Influence of Temperature

**Figure 3:**
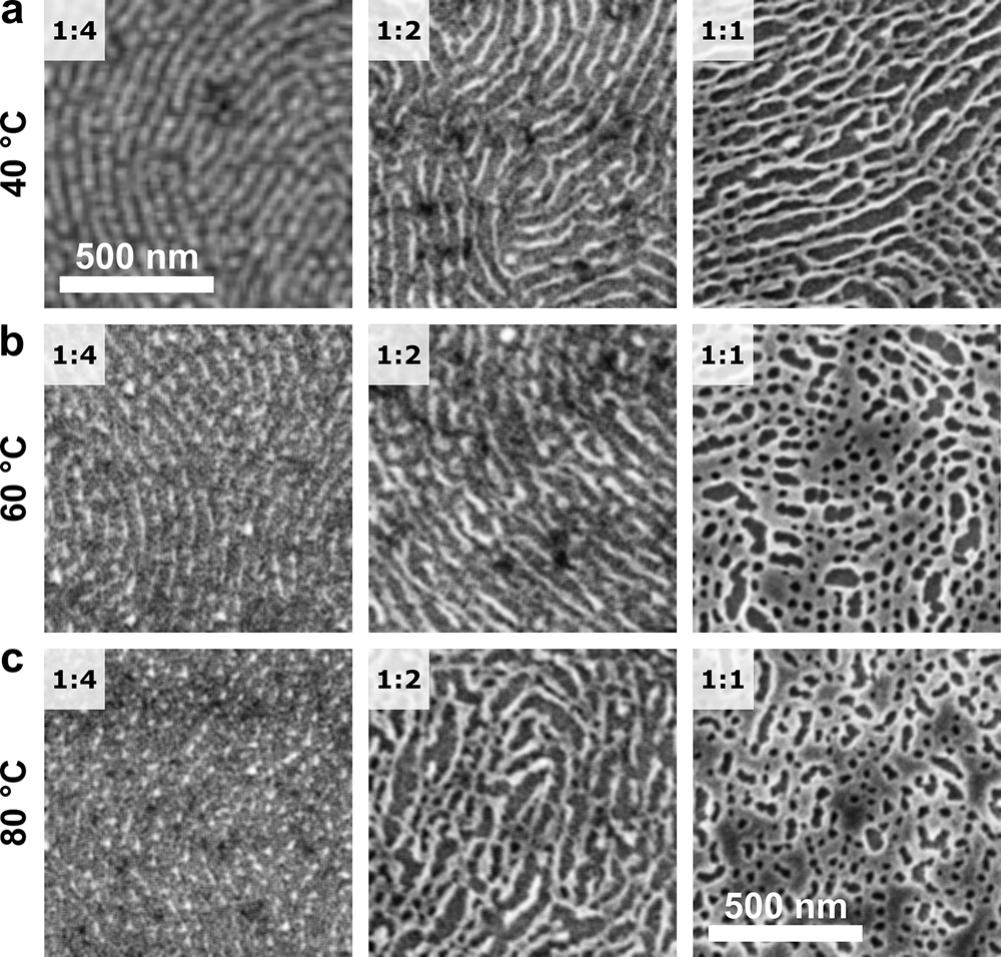
The morphologies of the cobalt oxide nanostructures obtained from cylindrical PS-*b*-P2VP C116 at different metal loadings (indicated as Co:VP ratios in the gray tabs), cast from a 1% BCP/10% TMOT–toluene mixture at elevated temperatures: (a) 40 °C, (b) 60 °C, and (c) 80 °C.

We observed the nonselective infusion and formation of inorganic surface deposits in the samples prepared by the rapid evaporation of the casting mixture at elevated temperatures. As illustrated by the behavior of the Co(acac)_3_–C116 system cast from the TMOT–toluene mixture in Figure 3 (for the other metals, see Figures S4–S6), the most efficient phase separation and ordering that yields continuous nanowires is, in general, achieved at low casting temperatures, i.e., 25–40 °C. Except for vanadium and manganese, the quality of the BCP morphology replication deteriorated when the Me(acac)_3_–BCP solutions were cast at elevated temperatures, gradually developing cross-branching at 60 and 80 °C for the Me:VP 1:2 metal loading and a perforated crust morphology at higher precursor concentrations. The relatively selective inclusion of the precursor into the P2VP block at 80 °C at lower loadings (1:2 Co:VP) could also indicate the kinetic origin of the observed disorder caused by the entrapment of the precursor in the nonpreferential polystyrene block during the rapid removal of the solvent at high temperature. Conversely, the high selectivity of the infusion of Mn(acac)_3_ and V(acac)_3_ into the P2VP block at elevated temperatures (Figures S5 and S6) and the observed lability (UV–vis data, Figure S1) of these complexes suggest stronger chemical interactions between the P2VP block and these precursors. Nonetheless, these successful patterning results indicate that, even in the case of V and Mn, the coordination of the polymer chains in the concentrated BCP solution is not strong enough to inhibit the BCP ordering by irreversible chemical cross-linking.

### Optimal Film Thickness

Conformal inorganic replicas of horizontal cylinder arrays are expected in thin films with thicknesses close to the native BCP’s periodicity (i.e., the cylinder-to-cylinder distance, which is ∼45 nm and ∼82 nm for C116 and C275, respectively).^58^ Here, high metal precursor loading can, in principle, affect the usual ordering landscape and swell the BCP in a selective or nonselective manner.^59^

**Figure 4:**
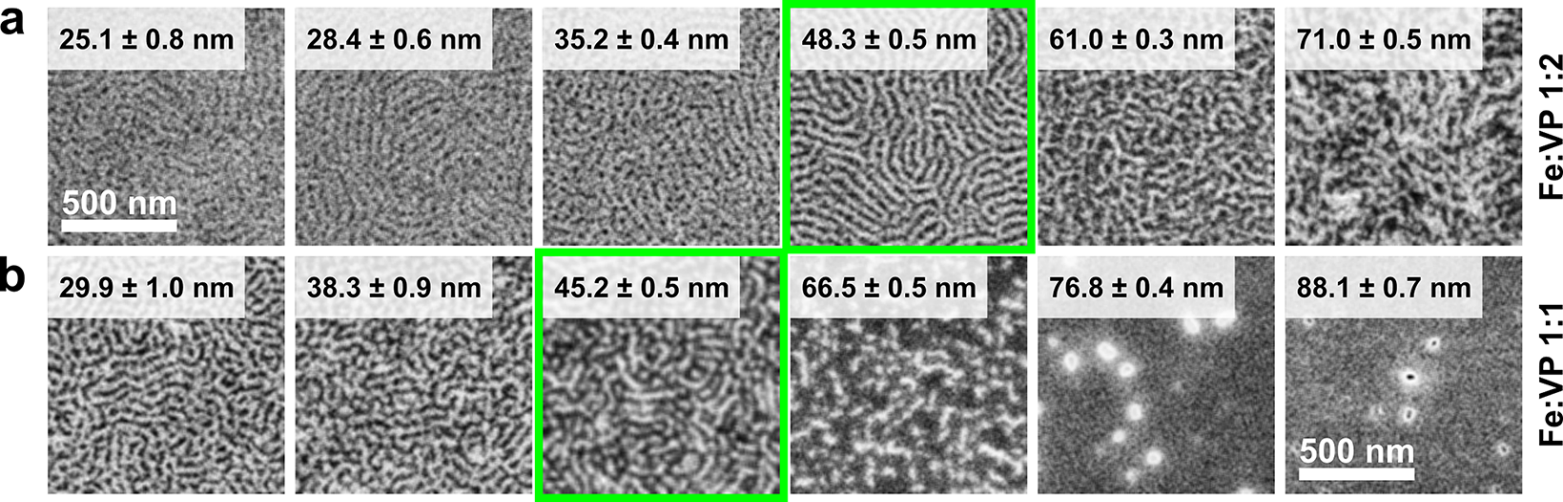
The evolution of the iron oxide replica with the thickness of the as-cast films of C116 at (a) 1:2 and (b) 1:1 Fe:VP metal loadings, along with the thickness measurement standard deviation values. The films yielding a horizontal monolayer of nanowires are outlined in green.

To test the evolution of the metal oxide nanostructures with the thickness of the as-cast films, we prepared a series of C116–Fe(acac)_3_ samples cast at various spin-coating speeds. The morphologies of the inorganic nanostructures that were obtained at the moderate 1:2 Fe:VP ratio follow a transition from monolayer cylinders to a bilayer mesh morphology in the films thicker than ≈70 nm (Figure 4a). We note that, at this metal loading, the weight fraction of Fe(acac)_3_ in the dry films is significant at 36% (29% v/v, assuming the bulk density of the precursor). Nevertheless, the morphology resembles that of a neat BCP, with the formation of double layers observed in the thicker films. At the higher 1:1 Fe:VP metal loading, the precursor becomes a major solid mass constituent of the system (53 wt % and 45 vol %, Figure 4b). Nanowire-like morphologies are observed at a thickness of ∼40 nm, which is 20% lower than in the neat BCP (Figure S2). At this loading, however, no discernible morphology can be observed in the thicker films that is indicative of nonselective precursor sequestration and inorganic surface shell formation.

### Composition of the Nanowires

**Figure 5:**
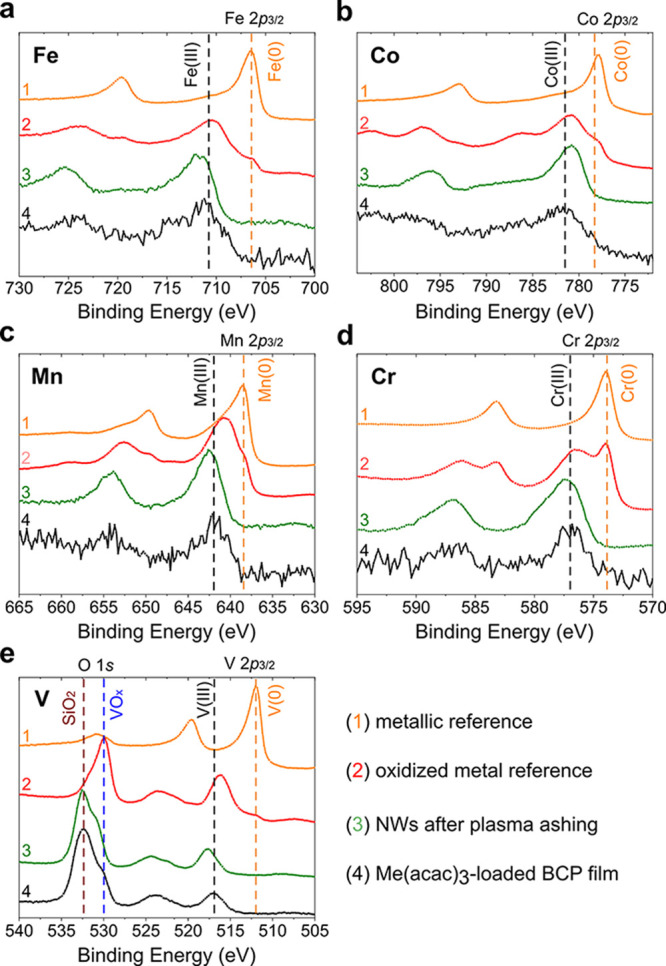
X-ray photoelectron spectra of the Me(acac)_3_-infused BCP templates at a 1:2 Me:VP metal loading ratio (black curves) and the inorganic nanowire replicas that were obtained after plasma ashing the organic material (green curves). Spectra of freshly cleaned metallic foils (orange curves) and oxidized metal surfaces (red curves) are shown as references. The vertical dashed lines mark the binding energy of the reference materials reported in the literature.^60^ Me = (a) Fe, (b) Co, (c) Mn, (d) Cr, and (e) V. The intensities of the XP spectra were normalized.

XPS measurements were used to confirm the presence of Fe, Co, Mn, Cr, and V and to determine their oxidation states in the obtained nanomaterials before and after the plasma removal of organic residues from the thin films. For each metal precursor, the following set of samples was prepared: the as-cast BCP film infused with the precursor (Me(acac)_3_-BCP), an inorganic BCP replica ashed with oxygen plasma (O_2_ plasma), and two reference samples. The first reference sample consisted of a high-purity metal foil that had its surface oxide layer mechanically removed by polishing with a series of sandpapers with increasing gradation before being freshly cleaned with Ar ion bombardment under a high vacuum. The second reference sample consisted of an air-oxidized metal foil containing native surface oxides, which acted as a reference for the metal oxides that spontaneously formed on the metal’s surface. Representative XPS spectra are presented in Figure 5.

In the Fe series, the XP spectra of the Fe(acac)_3_-infused polymer and the inorganic NWs after oxygen ashing (Figure 5a, black and green curves, respectively) are very similar in terms of their shape and Fe 2p peak positions. In particular, the Fe 2p_3/2_ binding energy (BE) is 711.8 eV for the oxygen plasma-cleaned sample and 711.2 eV for the iron(III) acetylacetonate–polymer hybrid thin film. Additionally, these spectra are similar to the spectrum of the oxidized Fe foil (Figure 5a, red curve; Fe 2p_3/2_ BE = 710.5 eV) in regard to their shape and the BE position of the Fe 2p peak, while they are significantly different from the reference spectrum of the metallic Fe foil (Figure 5a, orange curve; Fe 2p_3/2_ BE = 706.5 eV). This suggests that the Fe atoms in both the polymer hybrid and the NW samples have oxidation states similar to those of the Fe oxides that are produced by exposing Fe foil to air and that no metallic Fe is present in either of these samples. In terms of the oxidation states of the iron atoms, the Fe 2p_3/2_ peak for Fe_2_O_3_ is reported to have a BE of 710.8 eV and that of iron(III) oxide-hydroxide (FeO(OH)) has a BE of 711.8 eV;^60^ thus, we conclude that Fe(III) compounds are present in both the precursor-infused BCP and the as-synthesized inorganic nanowire replica.

**Figure 6:**
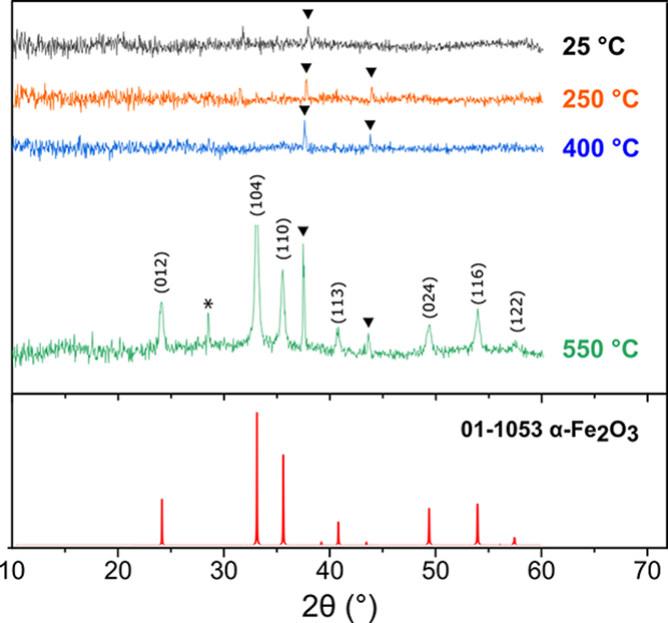
XRD patterns of the Fe(acac)_3_-infused C116 sample (Fe:VP of 1:1) cast on a silicon wafer from 10% TMOT in toluene after plasma ashing (black curve) and after being further annealed in air in a stepwise manner with 1 h dwells at 250, 400, and 550 °C (orange, blue, and green curves, respectively), with diffractograms registered at the end of each step with identified indices of the reflexes. The simulated diffraction pattern based on the α-Fe_2_O_3_ oxide CIF entry, along with the JCPDS card number, is shown at the bottom of the graph in red. The asterisks and triangles mark the reflexes originating from the silicon substrate and the silver heater, respectively.

Powder X-ray diffraction (PXRD) provides further information about the composition of the iron oxide nanowires. The NWs that were obtained by oxygen plasma ashing the Fe-containing BCP are amorphous or only contain very small-grained crystallites (Figure 6, black curve). To improve the crystallinity of the NWs, we annealed the sample by subjecting it to a thermal run consisting of three constant-temperature steps each lasting 1 h at 250, 400, and 550 °C. We collected *in situ* PXRD patterns at the end of each step. The material remains amorphous when heated at 400 °C (blue curve); however, after 1 h of annealing at 550 °C, the set of reflexes that are attributable to hematite (α-Fe_2_O_3_) are visible, which indicates crystallite coarsening. This trend, which was observed for an Fe(acac)_3_–BCP hybrid material, confirms the reports on the thermal decomposition of neat Fe(III) acetylacetonate, where α-Fe_2_O_3_ was reported as the dominant phase above 500 °C.^61^

We then performed analogous XPS and PXRD characterizations for the remaining transition metal nanostructures. The Co-containing samples display no significant differences between the XP spectra registered for the BCP–cobalt(III) acetylacetonate hybrid thin film and the NWs sample (Figure 5b). Additionally, the peak positions match those of the oxidized reference sample and those reported for Co(III) compounds.^60^ Conversely, the reference metallic Co 2p_3/2_ peak observed at 777.9 eV is significantly different than that for any of the other Co samples: 780.9 eV for the oxidized reference Co foil, 781.8 eV for the Co(acac)_3_–polymer hybrid, and 780.8 eV for the oxygen plasma-cleaned NWs. This result suggests that no metallic Co is present in the samples. A small difference in the BE of the Co 2p_3/2_ peaks between the Co(acac)_3_–BCP hybrid (781.8 eV; Figure 5b, black curve) and oxygen ashed sample (780.8 eV; Figure 5b, green curve) is observed and attributed to either a different composition of the polymer matrix for those two samples or experimental factors, such as noise level. PXRD data of the nanowires annealed in air at 550 °C for 1 h indicate that, even at elevated temperatures, the oxide remains amorphous (Figure S9d).

The trends for manganese (Figure 5c), chromium (Figure 5d), and vanadium (Figure 5e) are similar to those for the Fe- and Co-containing samples. In particular, the oxidation state of manganese in the Mn(acac)_3_–polymer hybrid before and after oxygen plasma ashing is most likely +3, as indicated by the similarities between the corresponding XP spectra and that of the oxidized metal foil reference.^60^ While the PXRD data of the unannealed manganese oxide immediately after the ashing step reveal that the material is amorphous, the onset of NW coarsening is the lowest in the tested series, with some crystalline reflexes appearing at 250 °C. Further annealing leads to the formation of Mn_2_O_3_ (orthorhombic, space group *Pbca*, #61; Figure S9a), which correlates well with the dominating oxidation state of the as-produced nanowires indicated by the XPS data.

In the case of Cr, oxygen plasma ashing caused a 1.5 eV upshift of the Cr 2p_3/2_ peak. It is also worth noting that the spectra for the oxide reference and the oxygen plasma ashed polymer hybrid thin film are more asymmetric, and the presence of an additional component at ca. 579–580 eV cannot be excluded (Figure 5d). Such an additional contribution and peak asymmetry were not observed for the Cr(acac)_3_–BCP blend, where only a Cr(III) component was present, which suggests that a portion of Cr was possibly oxidized to a higher oxidation state during the plasma ashing step. For example, the 2p_3/2_ BE peaks in Cr(VI) compounds have been reported between 579.5 and 580.2 eV.^60^ The PXRD measurements do not provide conclusive evidence for the presence of crystalline phases in the chromium oxide nanowires, both in the freshly ashed samples and after thermal annealing (Figure S9c).

Similarly for the vanadium series, the BE shift between the V(acac)_3_-infused BCP and the oxygen plasma ashed vanadium oxide nanowires (0.6 eV) might be related to the presence of a higher oxidation state of vanadium (e.g., V_2_O_5_) (Figure 5e). The presence of this oxide is evident in the powder diffractograms of divanadium pentoxide (orthorhombic, space group *Pmmn*, #59) nanowires that were collected on samples annealed at and above 400 °C (Figure S9b).

The binding energies of the 2p_3/2_ XPS peaks for all of the studied samples are summarized in Table S1. To conclude, the XPS and PXRD analyses for all of the investigated acetylacetonate–BCP blends confirm that the metallic precursor atoms remained at the original (+3) oxidation state and that the same state was dominant in the largely amorphous nanowires that were produced by oxygen plasma ashing the organic components, without the elemental metal species being present. The small BE shifts toward higher energies in the chromium and vanadium oxide NWs indicate the possible existence of minor components at higher oxidation states. Moreover, we note that, even in the case of high-temperature polymer ashing in a reducing atmosphere (600 °C, H_2_/Ar mixture), we did not observe the formation of metallic species (see the XPS data given in Figure S8), which has been previously observed for oxygen plasma ashed PS-*b*-P2VP after being infused with all-inorganic metal complexes (Fe(CN)_6_^3–^, Co(CN)_6_^3–^, CuCl_4_^2–^, and NiCl_3_^–^).^28^ However, since our samples were briefly exposed to air before being inserted into the XPS setup, we cannot eliminate the possibility of the rapid air oxidation of the as-produced NWs during their transfer to the high vacuum environment.

### Fabrication of the Volatile Organic Compound (VOC) Sensor

**Figure 7:**
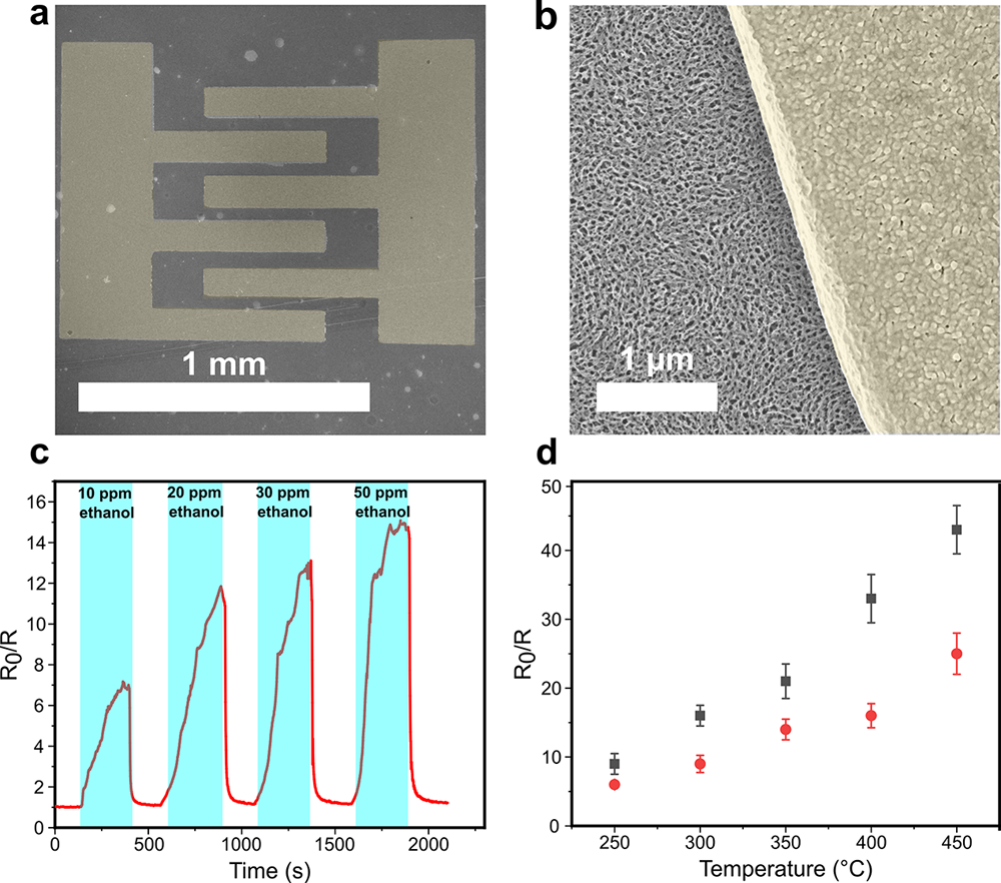
The C116-templated metal oxide nanowire gas sensor. (a) A false-color SEM image of the testing device having Au contact electrodes deposited on top of the porous (100 nm thick) metal oxide nanomesh. (b) A magnified SEM image that shows the boundary between the Au electrode and α-Fe_2_O_3_, which was annealed at 550 °C for 1 h. (c) The electrical response of the unannealed sensor to ethanol vapor, which was delivered in a stream of dry nitrogen at 10, 20, 30, and 50 ppm at 300 °C. (d) A comparison of the response (*R*_0_/*R*) of the unannealed sensor material (black points) and the sensor material after 1 h of annealing at 550 °C (red points) at different operating temperatures, measured 120 s after introducing the vapor into the test chamber. The reference ethanol concentrations that are indicated in (c) are specified within a ±15% confidence band due to the accuracy of the external sensor being subject to additional ±5% readout fluctuations. The error bars represent the StD of the signal readout within ±30 s from the central point value.

Transition metal oxide nanostructures are readily available for sensing applications due to their highly developed surface-to-volume ratio and exposure of reactive surface atoms to reactive adsorbates.^62^ In the past, BCPs have been successfully adapted as templates for the synthesis of nanopatterned sensing materials.^63,64^ Among the five metal oxide nanowires that we synthesized using a BCP template in this study, we decided to use iron oxide to demonstrate the facile fabrication of a proof-of-concept volatile organic compound (VOC) sensor and test its performance in ethanol vapor detection. In addition to conducting comprehensive examinations of the Fe_2_O_3_ sensor outlined in this study, we also evaluated the effectiveness of the remaining four transition metal oxides. Unfortunately, the outcomes of these tests were less encouraging (Figure S10). Fe_2_O_3_ is a common choice for volatile organic gas sensing^65^ due to several factors, including its thermal stability, environmental friendliness, and n-type semiconductivity.^65^ The sensing mechanism for α-Fe_2_O_3_, the most thermodynamically stable form of iron oxide, results from the existence of free electrons, which originate from the oxygen vacancies in this n-type semiconductor.^66^ At room temperature, free carriers are bound to the surface by the adsorbed oxygen and hydroxyl groups. At elevated temperatures and in the presence of adsorbed organic molecules, electrons are released from the conduction band, which causes the electrical resistance to decrease. Utilizing this operating principle, we constructed a functional sensing device as a proof of concept that consisted of a ∼120 nm thick iron oxide nanomesh (Figures 7 and S11), which was produced by four subsequent depositions of the Fe(acac)_3_–BCP mixture and plasma ashing steps (a detailed description of this process is given in the Experimental Section). We equipped the sensor with gold contact electrodes, which were thermally evaporated on top of the active material (Figure 7b). Then, we tested the device’s response to the presence of ethanol vapor in a stream of gas flowing through the chamber by measuring the ratio of the electrical resistance in the absence of ethanol (*R*_0_) to the resistance measured in the presence of ethanol (*R*). Figure 7c presents the response of the sensor to various ethanol concentrations in the 10–50 ppm concentration range. The low limit of detection is at the sub-1 ppm level, while the upper concentration testing level (≈100 ppm) was limited by the maximum flow of the ethanol vapor stream that was being fed to the gas mixer through a needle valve. The asymmetry in the time response, i.e., a slow rise time (o. 100 s) and a rapid fall time (o. 10 s), which was observed to a lesser extent with a commercial reference sensor, is a consequence of the slow diffusion and equilibration of the ethanol vapor delivery system and the relatively quick and efficient purge of the pure carrier gas at the end of each sensing cycle (Figure S12).

The magnitude of the response and the sensitivity of the sensor, i.e., *s* = (*R*_0_/*R*)/*c*_EtOH_, increases with the temperature at which the sensor operates. The sensitivity of the iron oxide NW sensor to ethanol in the range 250–450 °C is plotted in Figure 7d. Within the tested range, the sensitivity is linear, where *s* ≈ 0.32/ppm at 300 °C and *s* ≈ 0.18/ppm at 450 °C (Figure S13, Tables S2 and S3). Interestingly, the highest sensitivity is observed for the sensor that is composed of unannealed iron oxide nanowires; however, this occurs at the cost of having much higher electrical resistance values, which in turn provides a noisy response, especially at low temperatures (Figure 7d and Table S2). Thermal annealing at 550 °C, as shown by the PXRD measurements, induces crystallite coarsening in the nanowires and renders them more conductive. *R*_0_ is reduced from 150 to 37 MΩ when the sensor is operating at 300 °C (Tables S2 and S3), which also reduces the device’s recovery time. Annealing also potentially improves the interwire connectivity and thus the continuity of the current conduction pathway in the multilayered mesh. The cross-branching effect, which was discussed previously in the morphology characterization section, contributes to the increased specific surface area of the material, which is beneficial for catalysis and gas sensing applications. Based on the SEM measurements, we estimated the contour surface area of a single layer of the NWs to be ∼38 m^2^ g^–1^, which is a similar order to what has been reported for other nanostructured sensing materials.^67,68^ This is, however, the lower-bound estimate, as individual NWs are also internally porous. It should be noted that the production process of our prototype gas sensor material not only is straightforward but also delivers a sensor response that is comparable to, and sometimes even surpasses, the response of more intricate sensors, e.g., solution,^67^ hydrothermally grown,^68^ and solid-state^69^ synthesized nanostructured Fe_2_O_3_ sensing materials.

## Conclusions

In this study, we have demonstrated a one-pot, BCP-templated synthesis of transition metal oxide nanowires on silicon substrates by leveraging the interplay between the four components of the thin-film casting mixture: the macromolecular BCP template, the metal precursor, and a mixture of solvents that slows down the evaporation rate and enables BCP ordering to occur during the casting step. We established the optimal processing parameters that lead to the conformal replication of the BCP matrix morphology by appropriately tuning the constituent concentrations. Compared to the reactive (e.g., hydrolyzable or strongly complexing) precursors that are used for conventional two-step BCP patterning,^32,54^ Me(III) acetylacetonates are not strongly reactive toward the pyridine group in the 2-vinylpyridine molecules of the P2VP BCP block. Nonetheless, at the low and intermediate metal loadings of 1:4 and 1:2 Me(acac)_3_-to-vinylpyridine molar ratios, the precursor selectively sequesters into the P2VP domains, replicating the cylindrical morphology with a somewhat lower degree of lateral ordering than in the neat diblock. The low reactivity of the precursors enabled us to achieve a remarkably high metal loading; in the case of the 275 kg mol^–1^ cylindrical PS-*b*-P2VP, we were able to obtain a BCP-resembling inorganic nanomaterial morphology using a 1:1 Me:VP ratio, which corresponds to ∼50 wt % of the precursor in the dry mass of the film. Our temperature-resolved experiments show that metal loading is just one parameter that influences self-assembly. Both kinetics and thermodynamics also affect the precursor’s infusion selectivity. High-fidelity replicas of the BCP morphology for Fe, Co, and Cr were achieved at low casting temperatures and evaporation rates, allowing more time for preferred precursor–P2VP interactions. Elevated casting temperatures resulted in the formation of an inorganic material surface layer at the air–film interface for these metals. In contrast, the infusion of Mn(acac)_3_ and V(acac)_3_ was selective even at 80 °C. However, we could not currently assess the influence of removing the BCP template during plasma ashing on the final replica morphologies.

We also performed an XPS analysis and a PXRD study, which indicated that the presence of the amorphous Me(III) oxides in the as-produced inorganic nanowires largely resembles the XPS spectra of the precursor acetylacetonate compounds, irrespective of the BCP matrix removal method.

Finally, we used the developed metal oxide nanowire synthesis method to fabricate a gas sensor based on a multilayered porous iron oxide nanomesh; we then tested this gas sensor’s electrical response against ethanol vapor. Our approach further simplifies BCP-templated inorganic nanostructure fabrication by eliminating the conventional step of the BCP template ordering. The straightforward idea behind this approach, which relies on in-solution mixing, is easy to adapt as a platform for developing new materials, particularly for mixed-oxide nanomaterials.

## Supporting Information Available

UV–vis spectra of the Me–acetylacetonate solutions, which were recorded for evaluating their stability in the presence of excess pyridine molecules; SEM morphologies of the Fe-, Mn-, V-, Cr-, and Co-containing NWs, which were obtained under different casting conditions; a detailed description of the XPS analysis and the XP spectra of the films when subjected to thermal annealing in a reducing gas atmosphere; PXRD patterns for the other elements before and after thermal annealing; and further information on the performance of the gas sensor (PDF)
