# Supplementary material for: Block Copolymer-Templated, Single-Step Synthesis of Transition Metal Oxide Nanostructures for Sensing Applications
Source: ACS Appl Mater Interfaces. 2023 Aug 29;15(50):57970–80. doi: 10.1021/acsami.3c10439 (PMC10739603; doi:10.1021/acsami.3c10439)
Supplement: Supplementary file 1 — am3c10439_si_001.pdf [file am3c10439_si_001.pdf]

Supporting Information for:

Block Copolymer-Templated Single-Step Synthesis of Transition Metal Oxide Nanostructures for Sensing Applications

*Przemyslaw Pula[1], Arkadiusz A. Leniart[1], Julia Krol[1], Maciej T. Gorzkowski[2], Mihai C. Suster[3], Piotr Wróbel[3], Adam Lewera[1], Pawel W. Majewski[1]**

**Email: pmajewski@chem.uw.edu.pl*

[1]Department of Chemistry, University of Warsaw, Warsaw, 02093, Poland

[2]Biological and Chemical Research Centre, University of Warsaw, Warsaw, 02089 Poland

[3]Department of Physics, University of Warsaw, Warsaw, 02093, Poland

Contains:

Note 1 – UV-VIS spectra evolution of 5 mM Me(acac)$_3$ toluene solutions in the presence of 50 mM pyridine

Note 2 – Morphology characterization of metal oxide nanowires, supplementary SEM images, and FFT periodicity analysis

Note 3 – XPS calibration and analysis details

Note 4 – Supplementary PXRD patterns

Note 5 – Ethanol sensors' characterization details

Literature references

Note 1 – UV-VIS spectra evolution of 5 mM Me(acac)$_3$ toluene solutions in the presence of 50 mM pyridine

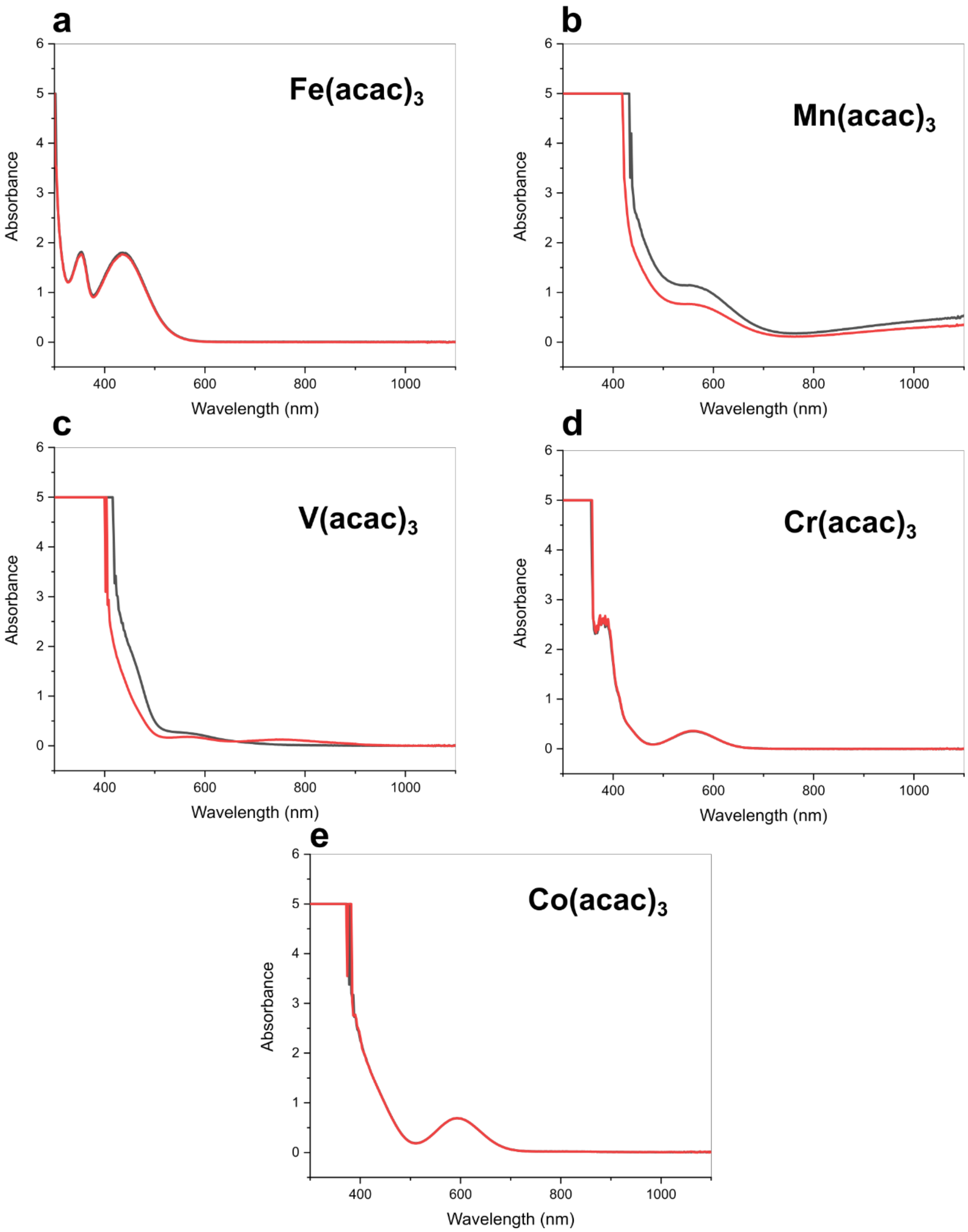

Figure S1. UV-VIS spectra of 0.2% (~5 mM) a) Fe, b) Mn, c) V d) Cr, e) Co acetylacetonate salt dissolved in toluene without (black curve) and after 24 hours after the pyridine addition (red line). Pyridine concentration in each solution was 50 mM corresponding to a 1:10 Me:pyridine molar ratio. Fe(acac)$_3$ solution was diluted 10 times before spectra collection to avoid detector oversaturation.

Note 2 – Morphology characterization of metal oxide nanowires, supplementary SEM images

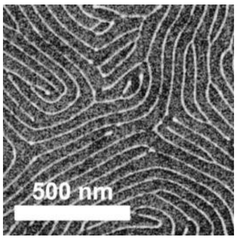

Figure S2. SEM image of PS-*b*-P2VP 116 kg mol^{-1} cast from 20% 3,4-dimethoxytoluene (DMOT)/ toluene solution and converted into an inorganic replica by further infusion with $Na_2[PtCl_4]$ platinum precursor, following the protocol by Buriak et al.[1]

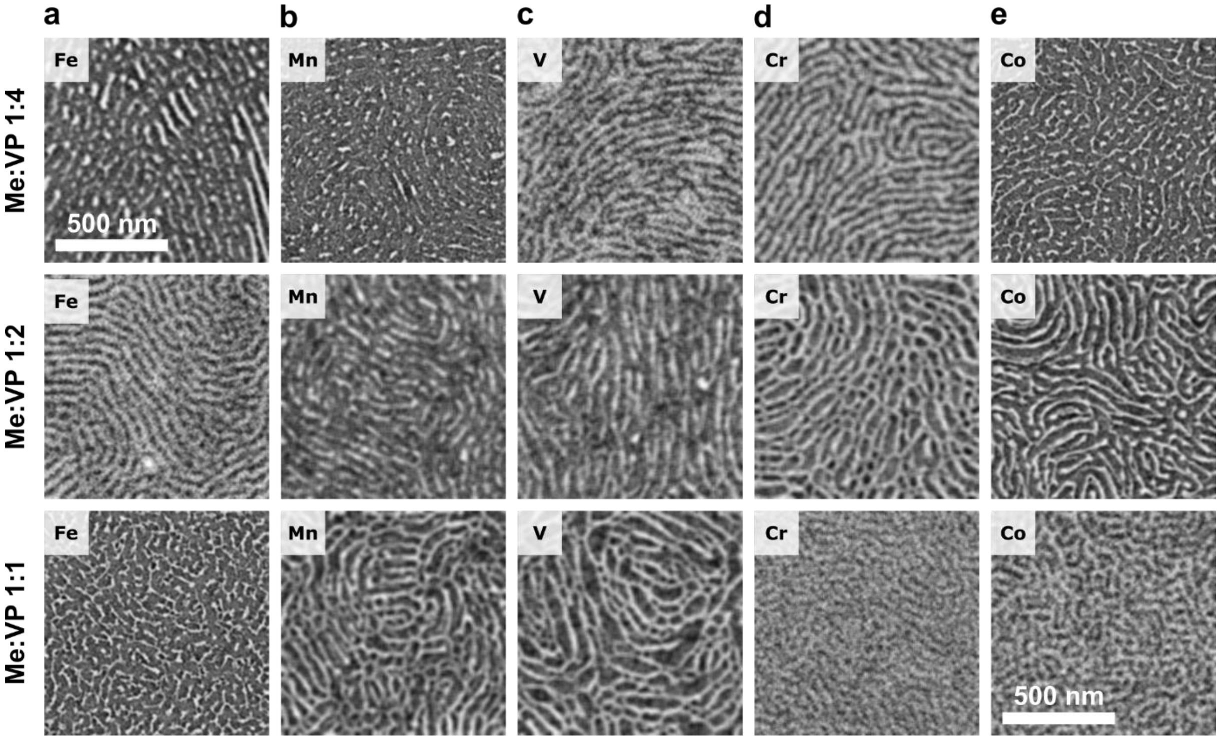

Figure S3. SEM morphologies of transition metal oxide nanostructures derived from cylindrical PS-*b*-P2VP 116 kg mol^{-1} (C116) blended with a) iron (III), b) manganese (III), c) vanadium (III), d) chromium (III), and e) cobalt (III) acetylacetonates at various Me:VP stoichiometric ratio. All samples were prepared by spin-casting from 1.0% BCP / 20% DMOT-toluene mixture at room temperature.

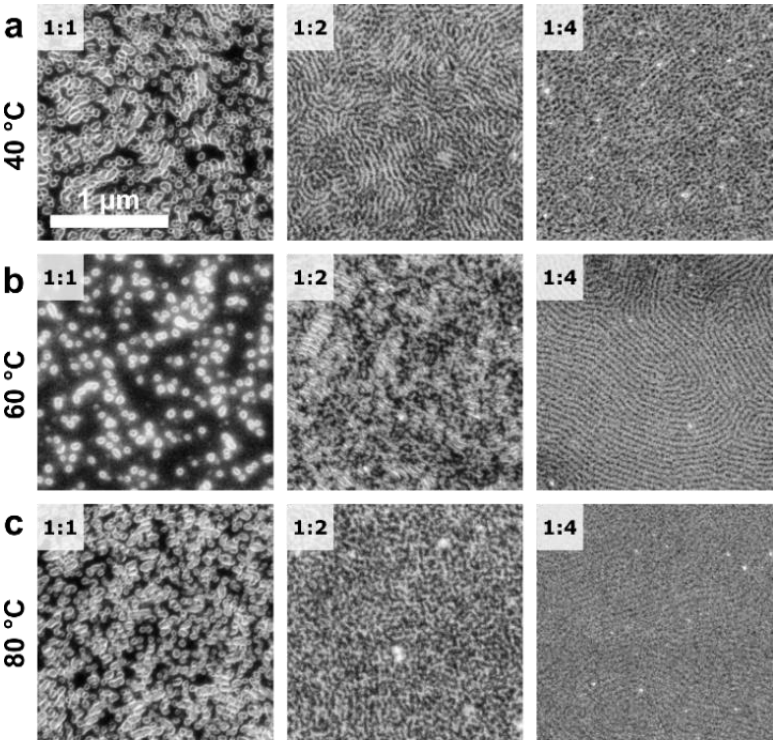

Figure S4. Morphology of **iron oxide** nanostructures obtained from cylindrical C116 PS-*b*-P2VP at different metal loading (indicated as Fe:VP ratio in grey squares) cast from 1.0% BCP / 10% TMOT-toluene mixture at elevated temperatures: a) 40 °C, b) 60 °C, and c) 80 °C.

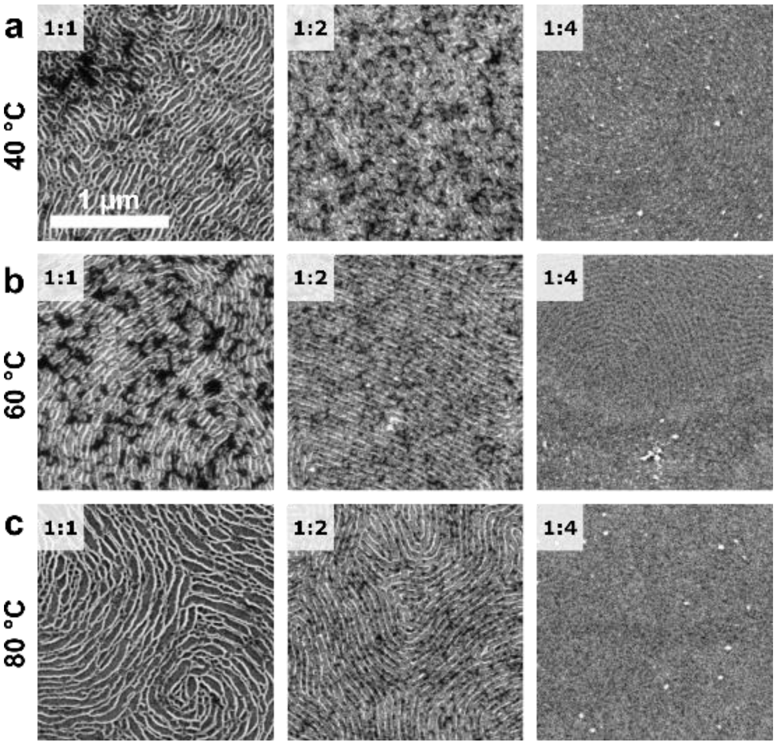

Figure S5. Morphology of **manganese oxide** nanostructures obtained from cylindrical C116 PS-*b*-P2VP at different metal loading (indicated as Mn:VP ratio in grey squares) cast from 1.0% BCP / 10% TMOT-toluene mixture at elevated temperatures: a) 40 °C, b) 60 °C, and c) 80 °C.

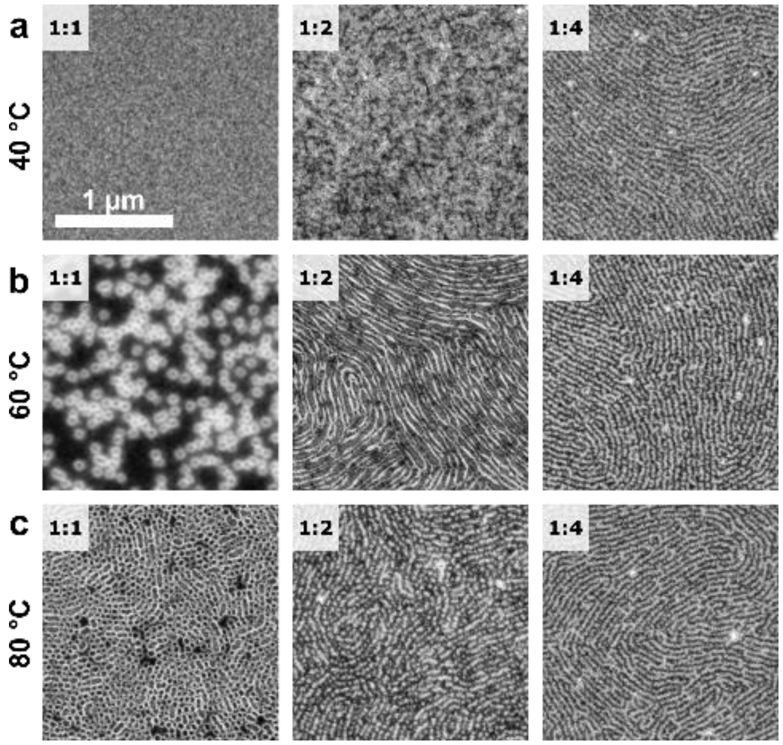

Figure S6. Morphology of **vanadium oxide** nanostructures obtained from cylindrical C116 PS-*b*-P2VP at different metal loading (indicated as V:VP ratio in grey squares) cast from 1.0% BCP / 10% TMOT-toluene mixture at elevated temperatures: a) 40 °C, b) 60 °C, and c) 80 °C.

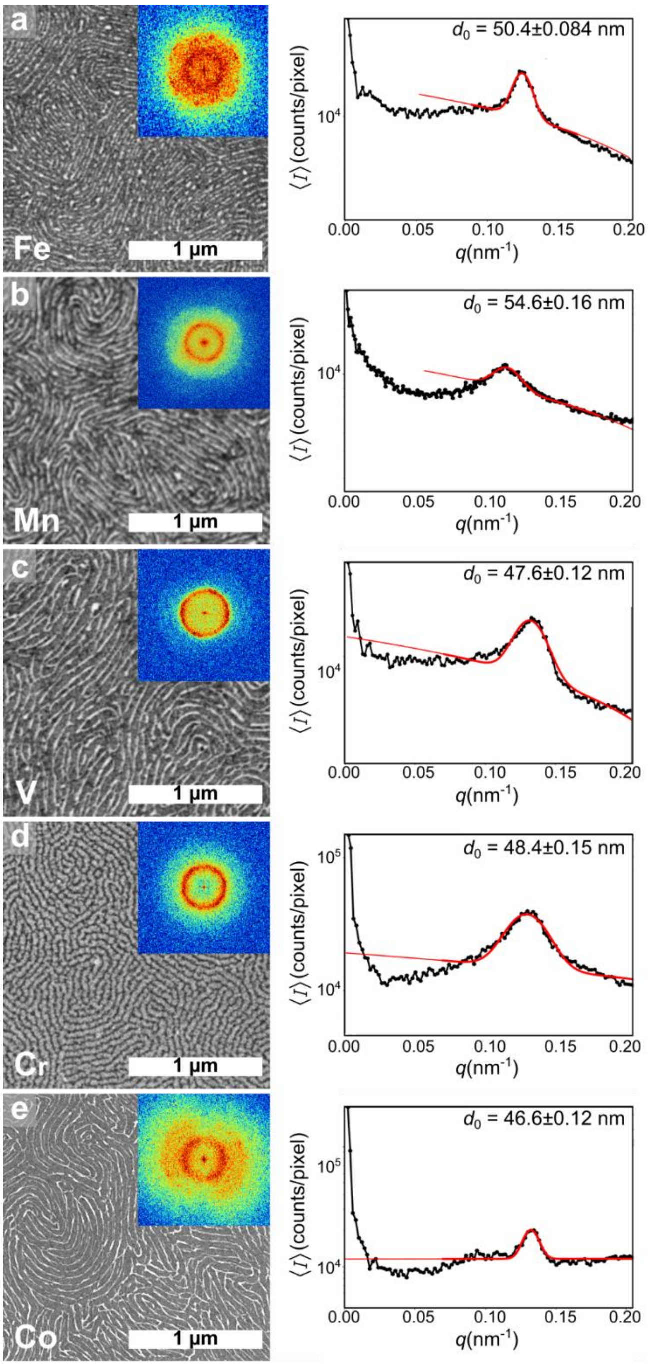

Figure S7. SEM morphologies of a) Fe, b) Mn, c) V, d) Cr and e) Co oxide nanostructures obtained from cylindrical C116 PS-*b*-P2VP at Me:VP 1:2 metal loading cast from 1.0% BCP / 20% DMOT-toluene mixture and their corresponding Fast Fourier Transforms (insets) used to calculate the the array periodicity (i.e., the average distance between the centers of the nanowires) (right). For the periodicity calculations, Python-written routines from the SciAnalysis package were adopted.[2]

Note 3 – XPS calibration and analysis details

In addition to oxygen plasma ashing described in the main text, we also tested two other methods of removal of polymer matrix: high-temperature ashing at 600 °C in the air in a furnace (*NWs air annealing at 600 °C*) and ashing at 600 °C in the rapid thermal processing (RTP) setup in the reductive atmosphere of a 5% H_2 in Ar i.e., reforming gas mixture, (*NWs after RTP annealing at 600 °C in H_2/Ar*). XPS spectra of the nanowires obtained using these ashing methods did not indicate the presence of metallic species. To properly determine the oxidation state of the metal deposits, we also registered the XP spectra of reference materials. In particular, for each patterned metal, we registered XP spectra of high-purity metallic foil polished and cleaned by Ar-sputtering, which were used as reference spectra of each metal in elemental form. We also registered XP spectra for the same foils partially oxidized by exposure to air. Representative spectra are presented in Fig. S8.

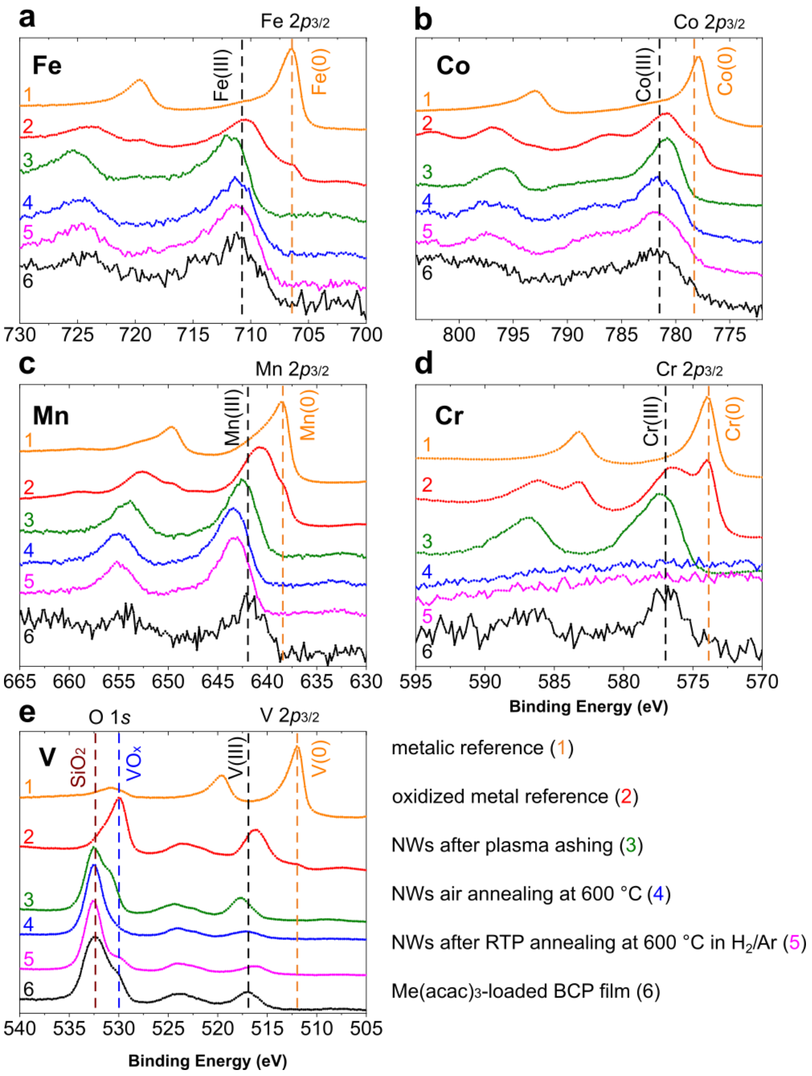

Figure S8. X-ray photoelectron spectra of Me(acac)$_3$-infused BCP templates at 1:2 Me:VP loading ratio (black curves) and inorganic nanowires replicas obtained by: plasma ashing of organic material (green curves), annealing in air at 600 °C (blue curves), and rapid thermal annealing at 600 °C in the reductive atmosphere (H_2/Ar) (pink curves). Freshly cleaned metallic foils (orange curves) and oxidized metal

surfaces (red curves) are shown as a reference. Vertical dashed lines mark the binding energy (BE) of respective signals for metal and polymer samples. a) Me = Fe, b) Co, c) Mn, d) Cr and e) V.[3]

Reference samples (those which did not contain polymer and metal precursors) did not require energy scale calibration. Other samples required careful energy scale calibration, especially because the adstructures deposited on silicon are not metallic and could undergo undesirable charging during the XPS measurement. Additionally, silicon wafers used as substrates were covered with a thin native silicon oxide layer that has lower conductivity than the doped silicon. Due to the presence of silicone oxide, two distinct Si 2*p* doublets were observed: at around 99 eV binding energy (BE), which were present in all investigated samples, attributed to a more conductive elemental Si substrate and an additional signal, whose position varied between samples, usually present between 103 and 107 eV. The latter signal was attributed to Si in a non-conductive (prone to charging during the XPS experiment) layer of SiO_2. As the investigated structures were deposited on top of that layer, the BE of Si 2*p* for the SiO_2 layer was used to calibrate the energy scale for all spectra collected for a given sample, using a reference value of 103.3 eV.[4] Charge neutralization was not used to avoid any possible changes in the oxidation state of the prepared materials.

Tab. S1 Summary of BE of $2p_{3/2}$ XPS peaks for elemental metal (*Metal reference*) and oxidized metal reference (*Oxide reference*) samples in comparison to $Me(acac)_3$-infused polymer (*$Me(acac)_3$-BCP*) and oxygen plasma ashed metal oxide nanowires (*NWs after oxygen plasma*).

BE (eV)	Fe $2p_{3/2}$	Co $2p_{3/2}$	Mn $2p_{3/2}$	Cr $2p_{3/2}$	V $2p_{3/2}$
Metal reference	706.5	777.9	638.6	573.9	512.0
Oxide reference	710.5	780.9	640.8	576.4	516.2
$Me(acac)_3$-BCP	711.2	781.8	641.9	576.9	517.0
NWs after oxygen plasma	711.8	780.8	642.8	578.4	517.6

Note 4 – Supplementary PXRD patterns

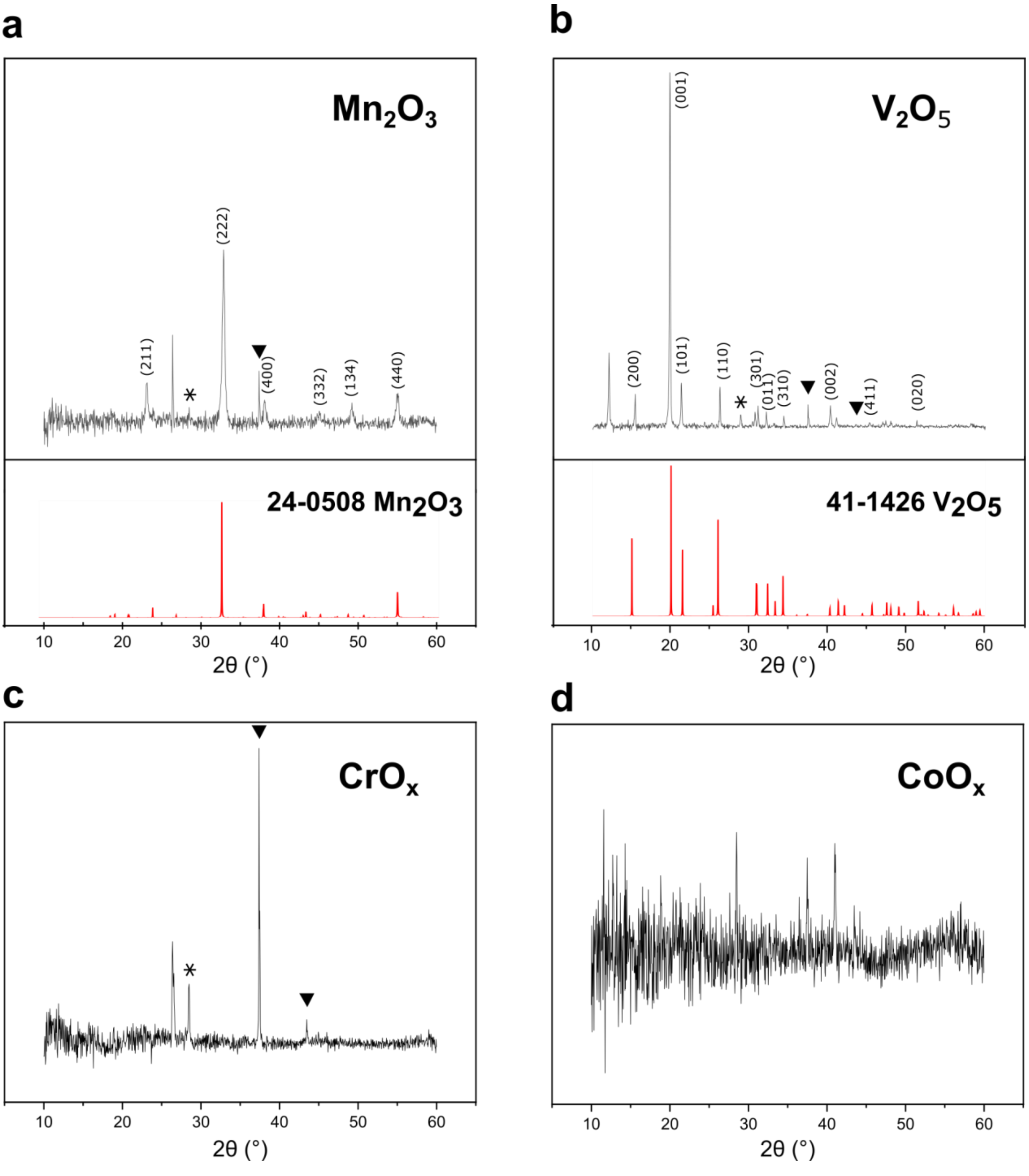

Figure S9. X-ray diffraction patterns of transition metal oxide nanowires obtained after ashing cylindrical C116 PS-*b*-P2VP-Me(acac)3 (films cast from 10% TMOT/90% toluene, infused with $Me(acac)_3$ at Me:VP 1:1 ratio) at 550 °C in air for 1h: a) Me = Mn, b) V, c) Cr, d) Co. For Mn and V oxides, the CIF generated diffraction patterns of the identified matching phases along with their JCPDS card numbers were included at the bottom of the graphs. Asterisks and inverted triangles represent peaks originating from the Si substrate and silver heater, respectively.

Note 5 – Ethanol sensors' characterization details

The resistance of a test device based on chromium oxide NWs was too high to be measured using electrical characterization setup used in this study.

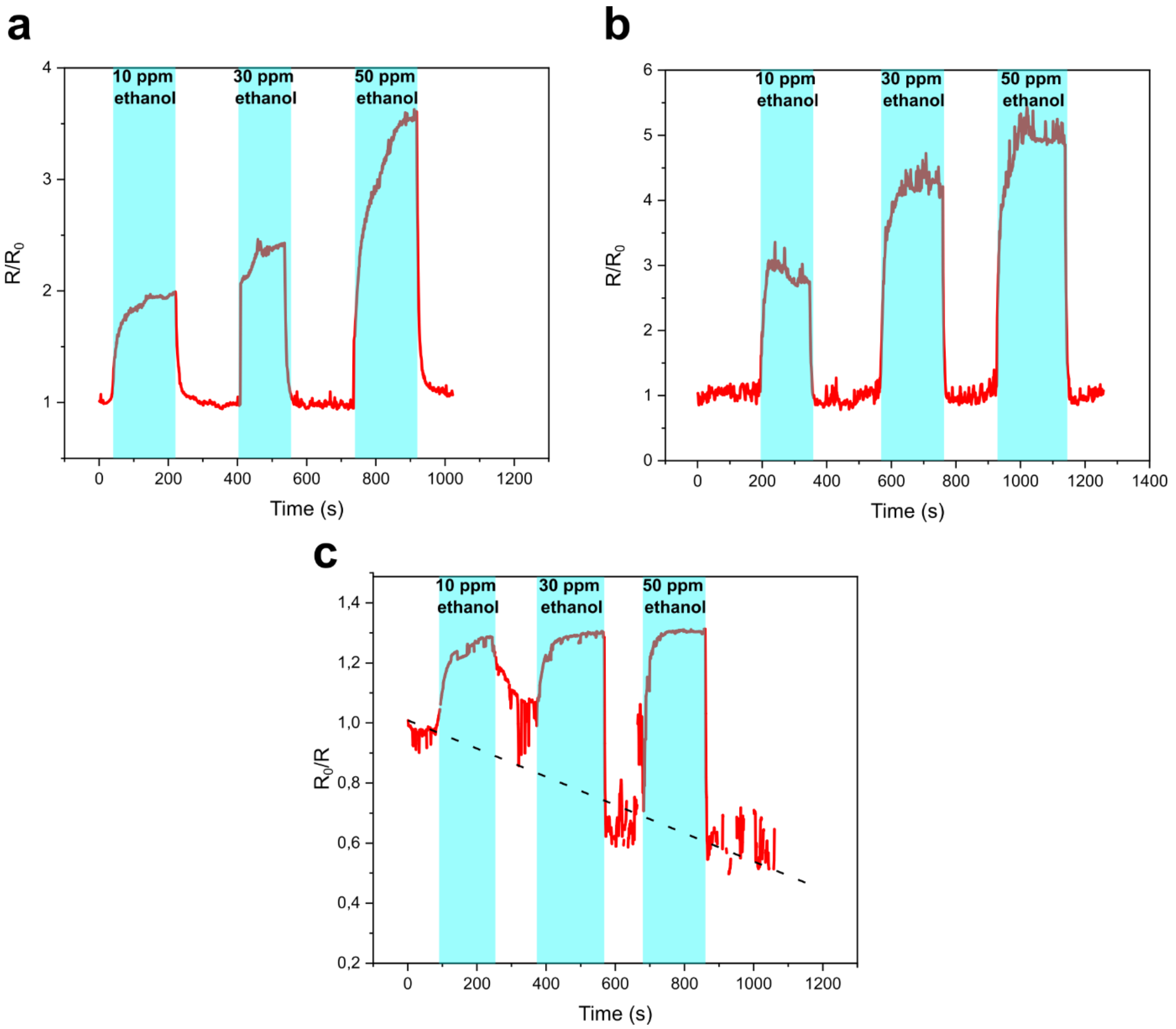

Figure S10. Electrical response of the a) manganese trioxide, b) cobalt oxide and c) vanadium pentoxide sensor to ethanol vapor delivered in a stream of dry nitrogen at 10, 30, 50 ppm measured at 300 °C. In V_2O_5 black dashed line marks the drift of the measured base resistance (R_0) resulting from a progressive metal-to-insulator transition at temperatures exceeding 280 °C.[5]

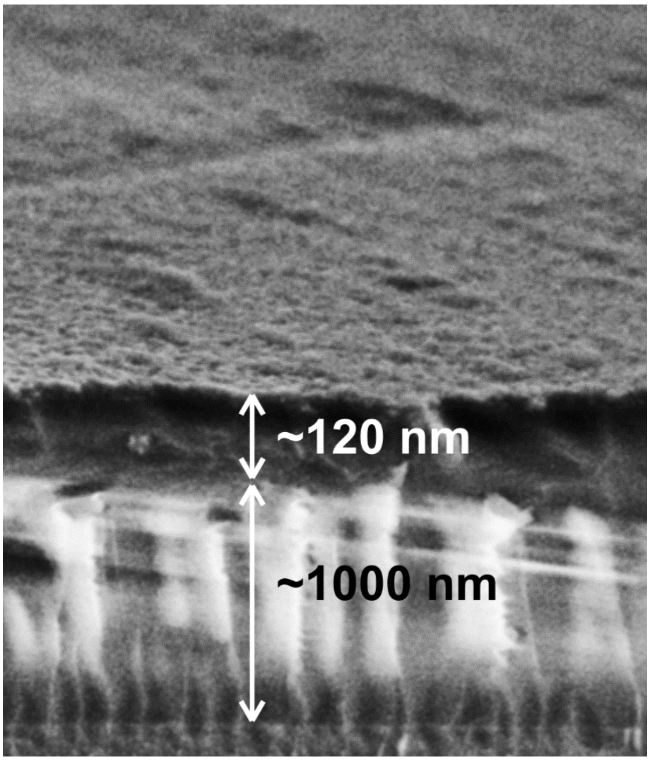

Figure S11. Cross-section image of ~120 nm thick iron oxide nanomesh after thermal annealing with indicated thickness of thermally grown SiO_2 (~1 μm).

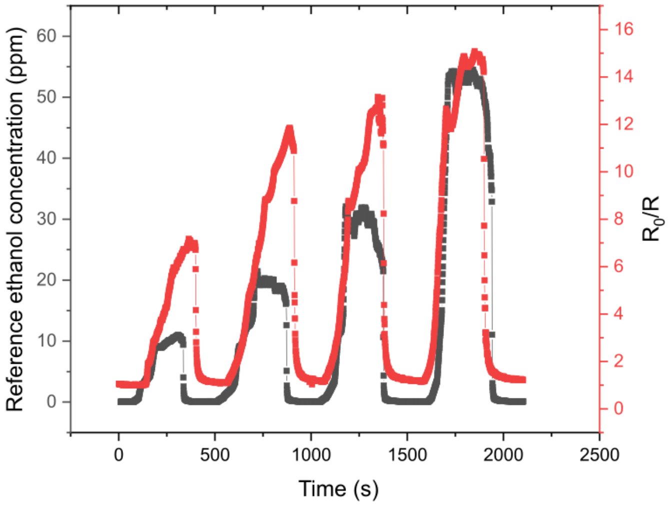

Figure S12. Sensor response to ethanol vapor delivered in a stream of dry nitrogen at 10, 20, 30, and 50 ppm at 300 °C (red symbols) with the reference concentration read-out values from the SGP-30 volatile organic compound gas sensor (black symbols).

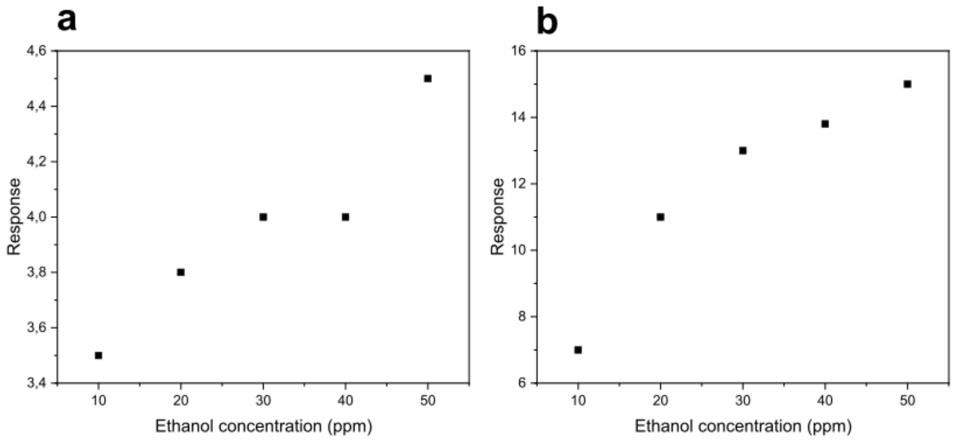

Figure S13. Sensor response, measured as R_o/R ratio, for ~120 nm thick iron oxide sensor annealed at 550 °C for 3 h operating at a) 300 °C and b) 450 °C.

Tab.S2. Electrical parameters of the iron oxide sensor before thermal annealing, an electrical response at the given temperature is reported after 150 s from the introduction of ethanol vapor at 50 ppm concentration.

**Operating temperature** (°C)	**Initial resistance, R_0** (MΩ)	**Electrical response** (R_0/R)	**Sensitivity** ($(R_0/R)/ppm_{EtOH}$)
250	230	9	0.18
300	150	16	0.32
350	43	21	0.42
400	30	33	0.66
450	13	43	0.86

Tab.S3. Electrical parameters of the iron oxide sensor after thermal annealing at 550 °C for 1h, an electrical response at the given temperature is reported after 150 s from the introduction of ethanol vapor at 50 ppm concentration.

**Operating temperature** (°C)	**Initial resistance, R_0** (MΩ)	**Electrical response** (R_0/R)	**Sensitivity** ($(R_0/R)/ppm_{EtOH}$)
250	60	6	0.12
300	37	9	0.18
350	22	14	0.28
400	13	16	0.32
450	8.5	28	0.56

Literature references

(1) Chai, J.; Buriak, J. M. Using Cylindrical Domains of Block Copolymers to Self-Assemble and Align Metallic Nanowires. *ACS Nano* **2008**, *2* (3), 489–501.

(2) Yager, K. G. SciAnalysis. http://gisaxs.com/index.php/Software (accessed Aug 09, 2023)

(3) *Handbook of X-Ray Photoelectron Spectroscopy: A Reference Book of Standard Spectra for Identification and Interpretation of XPS Data*; Moulder, J. F., Stickle, W. F., Sobol, P. E., Bomben, K. D., Chastain, J., King Jr., R. C., Physical Electronics, Incorporation, Eds.; Physical Electronics: Eden Prairie, Minn., 1995.

(4) Fairley, N.; Fernandez, V.; Richard-Plouet, M.; Guillot-Deudon, C.; Walton, J.; Smith, E.; Flahaut, D.; Greiner, M.; Biesinger, M.; Tougaard, S.; Morgan, D.; Baltrusaitis, J. Systematic and Collaborative Approach to Problem Solving Using X-Ray Photoelectron Spectroscopy. *Applied Surface Science Advances* **2021**, *5*, 100112.

(5) Kang, M., Kim, I., Kim, S. W., Ryu, J. W., Park, H. Y. Metal-insulator transition without structural phase transition in V2O5 film. *Applied physics letters* **2011**, *98*(13), 131907.
